# Association between maternal overweight or obesity and cerebral palsy in children: A meta-analysis

**DOI:** 10.1371/journal.pone.0205733

**Published:** 2018-10-16

**Authors:** Dongqiong Xiao, Yi Qu, Lan Huang, Yan Wang, Xihong Li, Dezhi Mu

**Affiliations:** 1 Department of Pediatrics, West China Second University Hospital, Sichuan University, Chengdu, China; 2 Key Laboratory of Birth Defects and Related Diseases of Women and Children, Sichuan University, Ministry of Education, Chengdu, China; University of Bristol, UNITED KINGDOM

## Abstract

**Context:**

There is no consensus regarding the association between maternal obesity or overweight and cerebral palsy (CP) in children.

**Objectives:**

To investigate whether maternal obesity or overweight is associated with CP and identify the factors that explain the differences in the study results.

**Data sources:**

We conducted a meta-analysis of studies published in English with titles or abstracts that discussed the relationships between maternal obesity or overweight and CP before August 23, 2017, using Ovid Medline, EMBASE and Web of Science.

**Study selection:**

Of 2699 initially identified studies, 8 studies that addressed the association between maternal obesity and CP met our final inclusion criteria.

**Data extraction:**

Information from the individual studies was abstracted using standardized forms by 2 independent observers who were blinded to the authors’ names and journal titles.

**Data synthesis:**

According to a random effects model, maternal overweight was significantly associated with CP in offspring [RR = 1.29 (95% CI, 1.04–1.60), heterogeneity (*I*^*2*^ = 45.5%, P = 0.103)]; maternal obesity was significantly associated with CP in offspring [RR = 1.45 (95% CI, 1.25–1.69), heterogeneity (*I*^*2*^ = 24.1%, P = 0.253)]; and maternal obesity III was significantly associated with CP in offspring [RR = 2.25 (95% CI, 1.82–2.79), heterogeneity (*I*^*2*^ = 0%, P = 0.589)]. However, maternal underweight was not significantly associated with CP in offspring [RR = 1.11 (95% CI, 0.88–1.38), low heterogeneity (*I*^*2*^ = 0%, P = 0.435)]. Factors that explained the differences in the meta-analysis results included study design, study location, and whether individual studies adjusted for potential confounders.

**Conclusion:**

This study suggests that maternal obesity and overweight increase the risk of CP in offspring. Further studies are required to confirm these results and determine the influence of variables across studies.

## Introduction

Obesity is becoming an epidemic health problem. This trend is particularly true for maternal obesity and overweight, which may be associated with adverse obstetric complications[1, 2]. Maternal obesity is associated with low Apgar scores at 5 minutes[2, 3], preterm birth[4], low birth weight, periventricular leukomalacia (PVL)[5], autism spectrum disorders (ASDs)[6, 7], and intellectual disability[8], which could alter the offspring’s neurodevelopmental outcomes[6]. Furthermore, maternal obesity is associated with an increased risk of gestational diabetes, hypertension, and preeclampsia, which may be associated with adverse neurodevelopmental outcomes[9]. In addition, maternal obesity is associated with type 2 diabetes, congenital anomalies[10], and asthma[11] in offspring. Maternal obesity and overweight are the leading cause of public health problems. More than half of women receiving prenatal care in the United States are overweight or obese[12], and maternal obesity and overweight are epidemic in Sweden[13] and other countries.

Cerebral palsy (CP), which leads to cognitive impairments and motor deficits[14], is multifactorial. CP occurs in two per 1000 live births in Sweden[13]. This disorder is a tremendous burden on society and families. Studies show that maternal chorioamnionitis[15], maternal preeclampsia[16], maternal age >35 years, a low Apgar score at 5 minutes, preterm birth[17], low birth weight, periventricular/intraventricular hemorrhage[18], and bronchopulmonary dysplasia (BPD) are associated with CP. Among recent studies focusing on the relationship between maternal obesity and CP in offspring[9, 12, 13, 17, 19–22], two studies showed that maternal weight during pregnancy is associated with CP in offspring[23, 24], and other studies indicated a significant association between maternal obesity and CP in children[9, 12, 13, 20, 21]. Maternal obesity may contribute to CP in offspring via maternal preeclampsia, preterm birth, or a low Apgar score at 5 minutes (asphyxia), as these parameters alter the uterine environment[25] and lead to maladaptive programming of the fetal brain[26]. Furthermore, maternal obesity may induce a chronic inflammatory state, such as chorioamnionitis, which may contribute to the development of CP[15]. However, two studies[12, 22] have reported that there is no significant association between maternal obesity and CP in children. The reason for this discrepancy may be that infants with CP whose mothers were not obese may have been affected by other factors that induce adverse neurodevelopmental outcomes.

To date, findings regarding this issue have been little known and inconsistent. Thus, we conducted a meta-analysis of human studies investigating the relationship between maternal obesity or overweight and CP in children.

## Methods

### Retrieval of studies

We searched Ovid Medline, EMBASE, and Web of Science for articles published before August 23, 2017. We used the following keywords and medical subject headings (MeSH) to search for the first theme: “overweight”, “over-weight”, “body weight”, “obes$”, “body weight changes”, “body fat distribution”, “body mass index”, “overnutrition”, “body weigh$”, “bodyweigh$”, “body mass$”, “bodymass”, “body fat$”, “bodyfat$”, “skinfold thickness”, and “waist-hip ratio”, using “OR” to connect relevant terms within the search field. For the second theme, we used “maternal”, “pregnancy”, “pregnan*”, “prenatal*”, “antenatal*”, “antepart*”, “gestat*”, “fetal development”, “transplacental exposure*”, “fetal programming”, “fetal growth”, “foetal development”, “gestational age”, “fetal age”, and “foetal age”, using “OR” to connect relevant terms within the search field. For the third theme, cerebral palsy, we used MeSH and key words such as “cerebral palsy”, “CP”, “spastic*”, “quadriplegi*”, “quadriplegia”, “tetraplegi*”, “diplegi*”, or “disabled children”. For the fourth theme, etiology, we used “risk”, “mortality”, or “cohort” to acquire the search results. We used “AND” to combine the first theme, the second theme, the third theme and the fourth theme (S1 Data). We restricted the search to human studies published in English. The identified studies were screened by reading the titles and abstracts, and two reviewers (Dongqiong Xiao and Lan Huang) subsequently read the full text of the remaining studies independently and then discussed any disagreements to reach a consensus.

### Definition

The weight categories were defined as follows: maternal overweight (25 kg/m^2^≤BMI<30 kg/m^2^), maternal obesity (BMI≥30 kg/m^2^), maternal obesity class I (30 kg/m^2^≤BMI<35 kg/m^2^), maternal obesity class II (35 kg/m^2^≤BMI<40 kg/m^2^), and maternal obesity class III (BMI≥40 kg/m^2^).

### Study selection criteria

The study inclusion criteria were as follows: (1) studies that evaluated the association between maternal overweight or obesity and the risk of CP in offspring; (2) a case-control or cohort study design; (3) studies that described the assessment of exposure and outcome; and (4) studies that reported hazard ratios (HRs) and the corresponding 95% confidence intervals (CIs), adjusted and/or unadjusted odds ratios (ORs) and 95% CIs, or adjusted and/or unadjusted relative risk (RR) estimates and 95% CIs for maternal overweight and obesity.

The exclusion criteria for the study were as follows: (1) duplicated studies, (2) review or meta-analysis articles, (3) case reports, (4) studies published in a language other than English, (5) animal experiment studies, (6) studies that did not examine the relationship between maternal obesity/overweight and CP in offspring, (7) studies with overlapping data, and (8) studies with unusable data.

### Data extraction

Data were independently extracted from the studies by two reviewers (Dongqiong Xiao and Lan Huang). The extracted data included the name of the first author; publication year; the country of the participants; study design; sample size; the method for assessing maternal obesity, overweight and CP; primary outcome; and adjusted confounders.

### Quality evaluation

The two reviewers (Dongqiong Xiao and Lan Huang) independently used the Newcastle-Ottawa scale (NOS)[3] to examine the methodological quality of all the included studies. The quality score was evaluated by assessing the study population selection (four items), comparability (one item), and the evaluation of exposure and outcome (three items). Studies with scores of at least 5 were deemed to be of high quality. Disagreements were resolved in the manner previously described.

### Statistical analysis

The included original studies used ORs, RR, or HRs to assess the association between maternal obesity or overweight and the risk of CP in offspring. Because the P0, which is the incidence of the outcome in the nonexposed group, was very small in our study and because RR = OR/((1-P0)+P0*OR)[27], RR≈OR; thus, the HR, RR and OR were directly considered in the RR[7], and we used ln(RR), ln(OR), and ln(HR) to combine these values. We pooled the RR across studies using the DerSimonian–Laird formula (random effects model)[28]. The statistical heterogeneity[29] of the studies was assessed by using *I*^*2*^. *I*^*2*^>50% and P<0.1 indicated high heterogeneity. A forest plot was used to display the RR and 95% CIs for each study, as well as the pooled RR and 95% CIs. We conducted subgroup analyses based on study design (case-control or cohort), study location (USA or other), time of maternal BMI measurement (pre-pregnancy or other), and adjusted confounding variables (maternal age, maternal race, child’s sex, maternal smoking status, and maternal diabetes status). We performed sensitivity analyses by omitting one study at a time. Publication bias was assessed with Egger’s[30] and Begg’s[31] tests. P<0.05 was considered statistically significant. The statistical tests were performed with Stata software (version 12).

## Results

### Literature search

**Fig 1:**
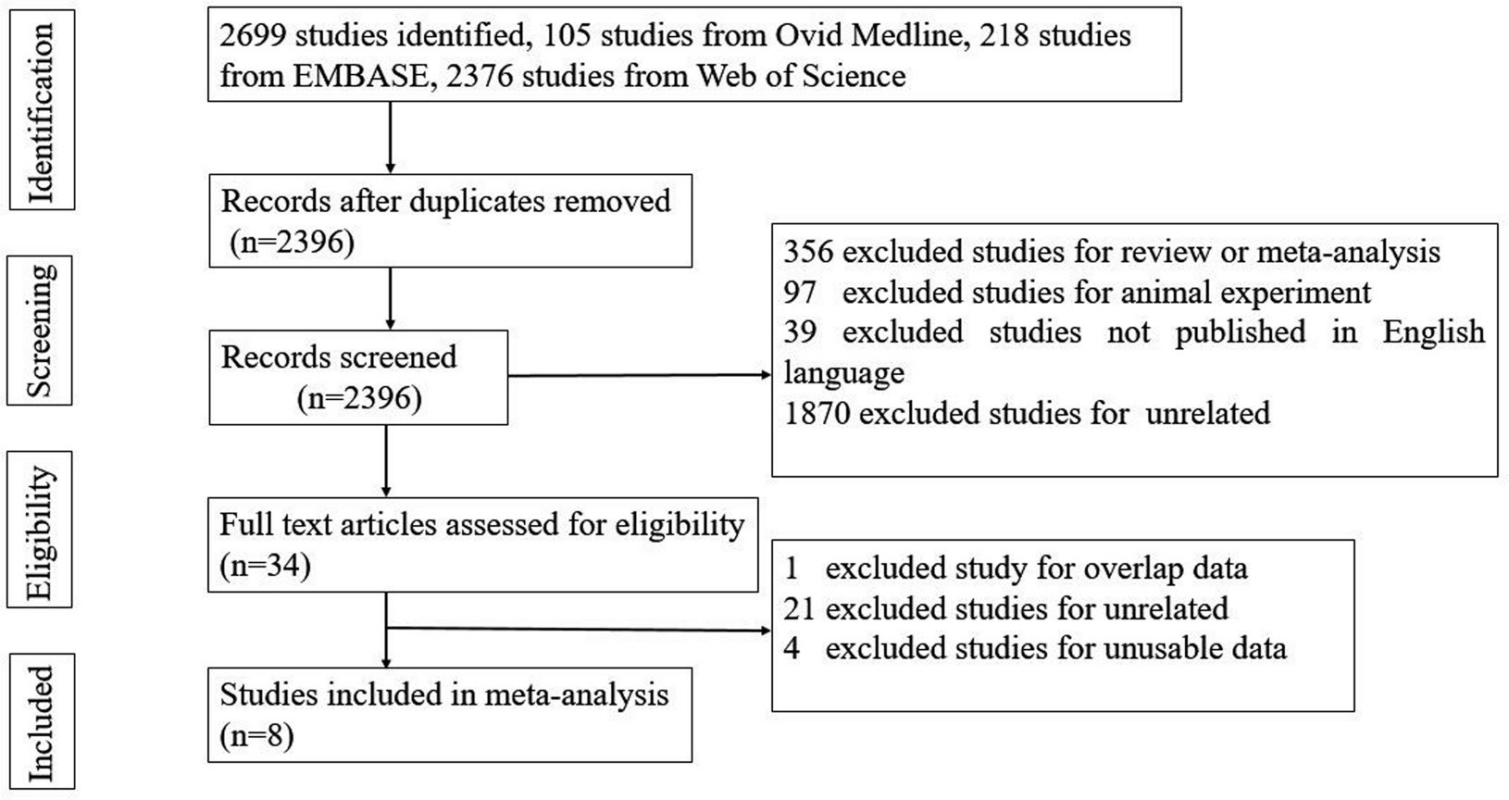
Flow chart for study selection.

We identified 2699 potential studies: 105 from Ovid Medline, 218 from EMBASE, and 2376 from Web of Science. After careful screening, 8 studies were selected for inclusion in this study (Fig 1). The extracted data from the 8 included studies are presented in Tables 1 and 2.

**Table 1:** Characteristics of the included studies.

Author, year	Country	Study design	Size	Time of BMI measurement	Ascertainment of exposure; outcome	Primary outcome	Adjusted confounding factors	Risk of bias; quality
Crisham Janik, 2013[19]	United States	Retrospective cohort	6221001	Prenatal or perinatal	Medical data; registry data	BMI≥30, OR = 1.27 (CI, 1.06–1.52) BMI≥40, OR = 2.56 (CI, 1.79–3.66)	Maternal race, maternal age, maternal education, no prenatal care, low insurance status, male infant	Different methods of exposure, only preterm neonates; NOS: 6
Pan, C, 2014[9]	United States	Retrospective cohort	83901	Pre-pregnancy	Medical data; registry data	BMI<18.5, OR = 0.46 (CI, 0.06–3.34); BMI = 25–29.9, OR = 1.15 (CI, 0.66–2.01); BMI = 30–34.9, OR = 1.62 (CI, 0.9–2.93); BMI = 35–39.9, OR = 2 (CI, 1–4.01); BMI≥40, OR = 2.95 (CI, 1.45–5.97)	Maternal age, education level, race, ethnicity; maternal intellectual disability; febrile at delivery; diabetic; hypertensive; tobacco user; sexually transmitted diseases; child’s sex, gestational age, birth weight	Different methods of exposure; NOS: 7
Forthun, I, 2016[20]	Norway, Denmark	Prospective cohort	188788	Pre-pregnancy	Self-reported; registry data	BMI<18.5, RR = 0.91 (CI, 0.5–1.67); BMI = 25–29.9, RR = 1.56 (CI, 1.21–2.01); BMI ≥30, RR = 1.55 (CI, 1.11–2.18)	Occupational status, smoking during the first part of pregnancy and age	Different methods of exposure; NOS: 7
McPherson,2016[12]	United States	Case-control	1669	Pre-pregnancy	Registry data	BMI = 30–39.9, OR = 1.1 (CI, 0.8–1.6); BMI ≥40, OR = 1.8 (CI, 0.9–3.4);	Preterm birth <28 weeks and magnesium sulfate exposure	Different methods of exposure, only preterm neonates; NOS: 6
Villamor, E, 2017[13]	Sweden	Retrospective cohort	1423929	Early pregnancy	Registry data	BMI<18.5, HR = 1.09 (CI, 0.84–1.41); BMI 25–29.9, HR = 1.22 (CI, 1.11–1.33); BMI 30–34.9, HR = 1.28 (CI, 1.11–1.47); BMI 35–39.9, HR = 1.54 (CI, 1.24–1.93); BMI ≥40, HR = 2.02 (CI, 1.46–2.79)	Maternal age, country of origin, education level, cohabitation with a partner, parity, height, smoking during pregnancy, and year of delivery	Different methods of exposure; NOS: 8
Love, E, 2012[22]	Scotland	Retrospective cohort	28967	Unknown	Registry data	BMI<20, OR = 0.99 (CI, 0.40–2.48); BMI = 25–29.9, OR = 0.81 (CI, 0.37–1.76); BMI>30, OR = 0.92 (CI, 0.35–2.47)	Maternal age, maternal smoking, gestational age, birthweight	Different methods of exposure; NOS: 7:
Nielsen, LF, 2008[29]	Denmark	Case-control	488	Pre-pregnancy	Medical records	BMI<18.5, OR = 1.7 (CI, 0.87–3.32); BMI = 25–29.9, OR = 0.9 (CI, 0.45–1.83)	Gestational age	Different methods of exposure; NOS: 6
Walstab, J, 2002[21]	Australia	Case-control	439	Pregnancy	Registry data; medical data	BMI = 25–29.9, RR = 3.48 (CI, 1.25–9.68)	No	Different methods of exposure; NOS: 6

CP, cerebral palsy; BMI, body mass index; RR, relative risk; OR, odds ratio; HR, hazard ratio; NOS, Newcastle-Ottawa score.

All primary outcomes were obtained after adjustment for several potentially confounding variables.

**Table 2:** Pooled results of the associations between maternal BMI and CP risk.

Variable	Underweight			Overweight			Maternal obesity (BMI≥30 kg/m^2^)			Obesity III (BMI≥40 kg/m^2^)		
	Studies	RR(95% CI)	*I*^*2*^(P-value)	Studies	RR(95% CI)	*I*^*2*^(P-value)	Studies	RR(95% CI)	*I*^*2*^(P-value)	Studies	RR(95% CI)	*I*^*2*^(P-value)
Total	4	1.11(0.88, 1.38)	0(0.435)	6	1.29(1.04, 1.60)	0.455(0.103)	6	1.45(1.25, 1.69)	0.241(0.253)	4	2.25(1.82, 2.79)	0(0.589)
Study location												
USA	1	0.46(0.06, 3.43)	NA	1	1.15(0.66, 2.01)	NA	3	1.47(1.09, 1.98)	0.603(0.08)	3	2.45(1.84, 3.27)	0(0.563)
Other	3	1.12(0.89, 1.40)	0(0.37)	5	1.31(1.01, 1.69)	0.560(0.059)	3	1.51(1.24, 1.83)	0(0.600)	1	2.02(1.46, 2.79)	NA
Study design												
Case-control	1	1.70(0.87, 3.32)	NA	2	1.68(0.45, 6.29)	0.781(0.033)	1	1.29(0.82, 2.03)	NA	1	1.8(1.93, 3.50)	NA
Retrospective cohort	3	1.05(0.83, 1.33)	0(0.624)	4	1.28(1.08, 1.51)	0.329(0.215)	5	1.48(1.24, 1.76)	0.374(0.172)	3	2.31(1.84, 2.90)	0(0.488)
BMI measurement												
Pre-pregnancy	3	1.14(0.66, 1.97)	0.255(0.261)	3	1.33(0.99, 1.80)	0.259(0.259)	3	1.63(1.27, 2.08)	0.2(0.287)	2	2.27(1.40, 3.68)	0(0.319)
Other	1	1.09(0.84, 1.41)	NA	3	1.34(0.76, 2.38)	0.607(0.079)	3	1.35(1.16, 1.56)	0.026(0.358)	2	2.25(1.77, 2.86)	0(0.336)
Adjustment factors												
Maternal age												
Yes	3	1.05(0.83, 1.33)	0(0.624)	4	1.28(1.08, 1.51)	0.329(0.215)	5	1.48(1.24, 1.76)	0.374(0.172)	3	2.31(1.84, 2.90)	0(0.488)
No	1	1.70(0.87, 3.32)	NA	2	1.68(0.45, 6.29)	0.781(0.033)	1	1.29(0.82, 2.03)	NA	1	1.80(0.9, 3.4)	NA
Child’s sex												
Yes	1	0.46(0.06, 3.43)	NA	1	1.15(0.66, 2.01)	NA	2	1.56(0.98, 2.49)	0.798(0.026)	2	2.63(1.91, 3.63)	0(0.726)
No	3	1.12(0.89, 1.40)	0(0.37)	5	1.31(1.01, 1.69)	0.560(0.059)	4	1.47(1.23, 1.76)	0(0.705)	2	1.98(1.48, 2.64)	0(0.76)
Maternal race												
Yes	1	0.46(0.06, 3.43)	NA	1	1.15(0.66, 2.01)	NA	2	1.56(0.98, 2.49)	0.798(0.026)	2	2.63(1.91, 3.63)	0(0.726)
No	3	1.12(0.89, 1.40)	0(0.37)	5	1.31(1.01, 1.69)	0.560(0.059)	4	1.47(1.23, 1.76)	0(0.705)	2	1.98(1.48, 2.64)	0(0.76)
Maternal smoking												
Yes	3	1.05(0.83, 1.33)	0(0.624)	4	1.28(1.08, 1.51)	0.329(0.215)	4	1.60(1.35, 1.91)	0.007(0.388)	2	2.16(1.61, 2.90)	0(0.34)
No	1	1.70(0.87, 3.32)	NA	2	1.68(0.45, 6.29)	0.781(0.033)	2	1.27(1.08, 1.50)	0(0.95)	2	2.37(1.73, 3.24)	0(0.36)
Maternal diabetes												
Yes	1	0.46(0.06, 3.43)	NA	1	1.15(0.66, 2.01)	NA	1	2.05(1.4, 3.0)	NA	1	2.95(1.45, 5.99)	NA
No	3	1.12(0.89, 1.40)	0(0.37)	5	1.31(1.01, 1.69)	0.560(0.059)	5	1.37(1.21, 1.55)	0(0.562)	3	2.19(1.75, 2.75)	0(0.521)

NA: not available

### Characteristics and quality of the included studies

The included studies were published between 2002 and 2017. All included studies were cohort and case-control studies. The sample sizes varied from a minimum of 439 to a maximum of 6,221,001 patients. In terms of the timing of maternal BMI assessment, four of the included studies[9, 12, 20,32] assessed maternal BMI during pre-pregnancy, one study[13] assessed maternal BMI during early pregnancy (<14 weeks)[13], and three studies[19, 21, 22] assessed maternal BMI at the hospital without mentioning the testing time. Five studies[9, 13, 21, 22, 32] used normal-weight (BMI 18.5–24.9 kg/m^2^) status as the reference, one study[20] used a lower normal weight (BMI 18.5–22.9 kg/m^2^) as the reference, and two studies[12, 19] used nonobese (BMI<30 kg/m^2^) participants as the reference. For the sources of the data used to determine maternal BMI, one study[20] used self-reported maternal weight and height, and seven studies used medical records or registry data. For CP diagnosis, all the studies identified cases from registry data or medical records. Some of the included studies statistically controlled for several potentially confounding variables, as shown in Table 1. The results of the quality assessment of the included studies are shown in Table 1. All the included studies were of high quality (NOS>5)(S1 Data).

### Maternal overweight or obesity and CP in children

**Fig 2:**
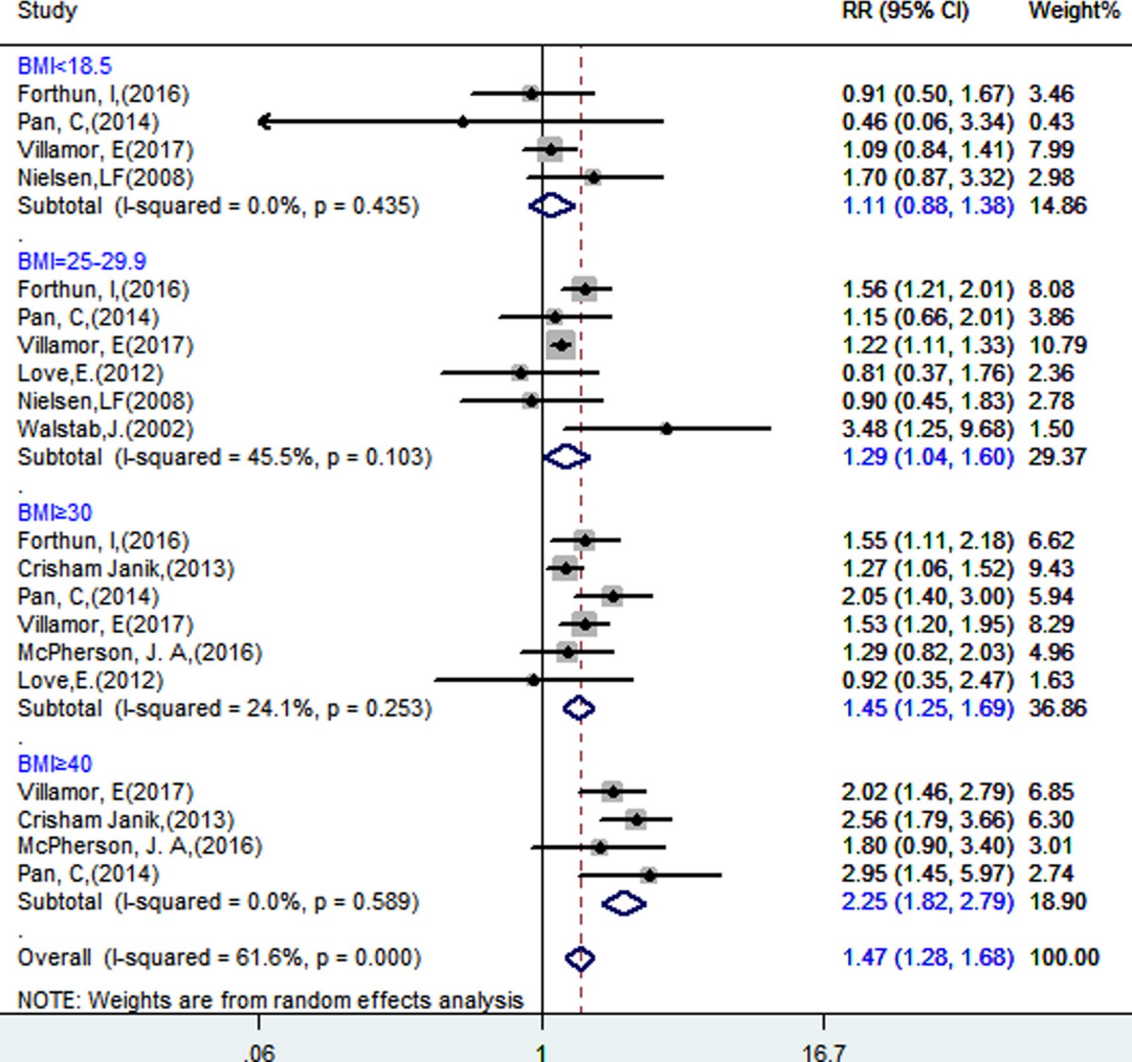
Forest plot of pooled analyses of maternal underweight, overweight, obesity or obesity III and CP in offspring, adjusted for several potentially confounding variables.

The original outcomes of the included studies are presented in Table 1 and Fig 2 shows the association between maternal BMI and CP risk in offspring: maternal underweight was investigated in 4 studies, with RR = 1.11 (95% CI, 0.88–1.38) and low heterogeneity (*I*^*2*^ = 0%, P = 0.435); maternal overweight was investigated in 6 studies, with RR = 1.29 (95% CI, 1.04–1.60) and heterogeneity (*I*^*2*^ = 45.5%, P = 0.103); maternal obesity was investigated in 6 studies, with RR = 1.45 (95% CI, 1.25–1.69) and heterogeneity (*I*^*2*^ = 24.1%, P = 0.253); and maternal obesity III was investigated in 4 studies, with RR = 2.25 (95% CI, 1.82–2.79) and heterogeneity (*I*^*2*^ = 0%, P = 0.589). The results from each study were adjusted for several potentially confounding variables, as shown in Table 2.

### Stratified analysis and sensitivity analysis

A stratified analysis was conducted to investigate possible sources of heterogeneity in studies of maternal BMI and CP risk in offspring (Table 2). The association between maternal underweight and CP in offspring was nonsignificant, and this association was consistent when stratified by different factors. The association between maternal obesity III and CP in offspring was statistically significant, and this association was consistent when stratified by different factors.

The association between maternal overweight and CP in offspring was inconsistent when stratified by different factors. Stronger associations between maternal overweight and CP in offspring were found in countries other than the USA. Stronger associations between maternal overweight and CP in offspring were found in retrospective cohort studies than in case-control studies. When the results were stratified by certain cofounding variables, stronger associations were found when adjustments were made for maternal age and maternal smoking status than when these confounding variables were not adjusted, while stronger associations were found without adjustments for the child’s sex, maternal race, and maternal diabetes status than when these confounding variables were adjusted.

The association between maternal obesity and CP in offspring was consistent when stratified by study location, BMI assessment, maternal smoking and maternal diabetes. Stronger associations between maternal obesity and CP in offspring were found in retrospective cohort studies than in case-control studies. When the results were stratified by certain confounding variables, stronger associations were found with adjustments for maternal age than without adjustments for maternal age; however, stronger associations were found when the child’s sex and maternal race were not adjusted than when these parameters were adjusted.

Considering the small number of studies in our meta-analysis, we omitted one study at a time to perform a sensitivity analysis. For underweight patients and maternal obesity class III patients, no heterogeneity was observed during the sensitivity analysis. For overweight patients, when the study by Walstab et al[21] was omitted, the heterogeneity decreased (*I*^*2*^ = 24.3%, P = 0.259). For maternal obesity patients, when the study by Crisham et al[19] was omitted, the heterogeneity decreased (*I*^2^ = 0%, P = 0.434).

### Publication bias

Publication bias tests and plots were conducted when the meta-analysis included more than 10 studies; however, these tests were underpowered to detect publication bias when there were only 8 studies included in the meta-analysis (S1 Data).

## Discussion

To our knowledge, this study is the first meta-analysis of the relationship between maternal BMI and the risk of CP in offspring. The results of this meta-analysis, which included 8 studies with a total of 7,949,182 participants, showed that maternal overweight and obesity were significantly associated with CP in children. Maternal overweight, maternal obesity, and maternal obesity III were associated with a 29%, 45%, and 125% higher risk of CP in offspring, respectively. Maternal underweight was not associated with CP in offspring.

Stronger associations between maternal obesity and CP risk were found in our meta-analysis, especially in the USA and Europe. Maternal obesity has increased globally in recent years, and the incidence of adverse outcomes is increasing[33]. The number of women with maternal BMI ≥35 kg/m^2^ doubled from 50 to 100 million between 2000 and 2010[34]. The etiology of maternal obesity may be related to maternal age[13], race[17], and lifestyle. These findings may provide motivation for women to maintain an appropriate weight before or during pregnancy. Maternal obesity is significantly related to Apgar scores <7 at 5 minutes[3], preterm delivery, periventricular leukomalacia (PVL)[35], and neurodevelopmental disability[36], which may contribute to CP. Obesity may induce inflammation[37], which may increase the risk of CP. Maternal infections diagnosed during pregnancy were associated with an increased risk of CP in offspring in a study by Bear et al[17]. Maternal chorioamnionitis and obesity increased the risk of PVL beyond that expected for preterm neonates[35].

Maternal obesity might affect fetal neurodevelopment through multiple pathways. Maternal obesity has an impact on fetal anomaly screening[38] and birth defects[39]. Maternal obesity is associated with neurodevelopmental and psychiatric disorders in offspring[40–44] via related alterations in the uterine environment[25] and/or epigenetic processes[26, 45]. The mechanisms through which maternal obesity alters the neurodevelopment of offspring may be related to placental inflammation, lipotoxicity, and oxidative stress, and these changes in the uterine environment may contribute to maladaptive programming of the fetal brain. Another mechanism by which maternal obesity may affect the neurodevelopment of offspring may be related to insulin resistance, which contributes to abnormal central glucose metabolism and transport. One study[46] analyzed the whole placental transcriptome in obese and normal-weight women and showed that maternal obesity had a negative effect on placental development and function. Another study[47] used oxidative stress biomarkers to analyze samples from mothers and offspring and found that maternal obesity could affect the maternal microenvironment and that oxidative stress has a negative impact on the placenta and fetal growth. Brain-derived neurotrophic factor (BDNF) is necessary for placental development and fetal growth. An additional study[48] found that pre-pregnancy/early maternal obesity adversely affects BDNF signaling and affects placental function and fetal growth. A study by Hatanaka et al[49] showed that maternal obesity leads to the abnormal development of the neuronal circuitry and the loss of synapses. A study by Niculescu et al[50] demonstrated that maternal obesity may alter fetal hippocampal development and that maternal exposure to a high-fat diet induces fetal resorption and small-for-gestational age (SGA) offspring. A study[51] by Tozuka et al demonstrated that maternal obesity impairs hippocampal progenitor cell division and neuronal production in young offspring. Thus, the association between maternal obesity and CP risk in children may be mediated by various factors.

The association between maternal overweight and obesity and CP in offspring was inconsistent when the results were stratified by study location, study design, and certain confounding variables. Maternal overweight and obesity could contribute to CP in children via multiple factors, including study location, study design, maternal age, maternal race, maternal smoking status, maternal diabetes status, and child gender. The heterogeneity decreased when the study of Pan et al was omitted, a result that may be related to the adjustment for maternal diabetes. In addition, the source of heterogeneity may be the factors in the stratified analysis that were mentioned above. Although the meta-analysis adjusted for several confounding variables, potential biases due to other factors that contribute to childhood CP cannot be excluded. Other factors may interact with maternal obesity along the pathogenetic pathway that leads to CP in offspring.

This meta-analysis has several limitations. First, we may have missed some studies because we included only studies that were published in English and obtained from the Ovid Medline, EMBASE, and Web of Science databases. Furthermore, the pooled results of this meta-analysis should be interpreted with caution because they depend on a small number of studies, and the publication year may be a source of bias. Second, quantitative synthesis cannot eliminate the biases inherent to observational studies. Third, it is very difficult to visually assess publication bias when the number of included studies is only 8. Fourth, meta-analytic methods introduce limitations, especially when the source of heterogeneity is unclear or when publication bias is present. Although we did not find statistical evidence of publication bias, there may be publication bias, errors in data abstraction and incomplete ascertainment of published studies.

One of the major advantages of our study is that most of the included original articles used a cohort design, which excludes the possibility of reverse causation. Additionally, the sample size was large, which enhanced the statistical power for precise and reliable estimation.

In conclusion, our pooled analyses provide evidence that maternal obesity and overweight are significantly associated with CP in offspring. Additional studies are required to further investigate this question by identifying more risk factors.

## Supporting information

S1 Checklist PRISMA checklist. (DOC)

S1 Data Retrieval strategy, New Castle Ottawa(NOS) quality assessment for included. (DOC)
