# Supplementary material for: Association between maternal overweight or obesity and cerebral palsy in children: A meta-analysis
Source: PLoS One. 2018 Oct 16;13(10):e0205733. doi: 10.1371/journal.pone.0205733 (PMC6191132; doi:10.1371/journal.pone.0205733)
Supplement: S1 Data — (DOC) [file pone.0205733.s002.doc]

**Association between Maternal Overweight or Obesity and Cerebral Palsy in Children: A Meta-Analysis**

**Supplement 1. Retrieval strategy of Ovid Medline, EMBASE and Web of Science**

Before 23 August 2017.

**OVID**

1 exp Overweight/

2 Body Weight/

3 (obes$ or overweight or over-weight).tw.

4 exp Body Weight Changes/

5 (weight adj2 (loss or lost or losing or reduc$)).tw.

6 (weight adj2 (gain$ or increas$)).tw.

7 exp body fat distribution/ or body mass index/ or skinfold thickness/ or waist-hip ratio/

8 (body weigh$ or bodyweigh$ or body mass$ or bodymass or body fat$ or bodyfat$).tw.

9 Overnutrition/

10 (overeat$ or over-eat$ or overnourish$ or over-nourish$ or overnutrit$ or over-nutrit$).tw.

11 or/1-10

13 exp Overweight/

14 Body Weight/

15 (obes$ or overweight or over-weight).tw.

16 exp Body Weight Changes/

17 (weight adj2 (loss or lost or losing or reduc$)).tw.

18 (weight adj2 (gain$ or increas$)).tw.

19 exp body fat distribution/ or body mass index/ or skinfold thickness/ or waist-hip ratio/

20 (body weigh$ or bodyweigh$ or body mass$ or bodymass or body fat$ or bodyfat$).tw.

21 Overnutrition/

22 (overeat$ or over-eat$ or overnourish$ or over-nourish$ or overnutrit$ or over-nutrit$).tw.

23 or/13-22

24    11 and 23

25 (risk or mortality).mp. or cohort.tw.

26 24 and 25

27 exp Pregnancy/ or pregnancy.mp

28 fetal development.mp or exp fetal development/

29 (fetal development or pregnancy or pregnan* or maternal or transplacental exposure* or gestat* or fetal development or fetal programming or fetal growth or foetal programming or foetal growth or gestational age or fetal age or foetal age or prenatal* or antenatal* or antepart*).tw.

30 27 or 28 or 29

31 26 and 30

EMBASE

1 Cerebral Palsy/

2 cerebral palsy.ti,ab.

3 CP.ti,ab.

4 spastic*.ti,ab.

5 quadriplegi*.ti,ab.

6 Quadriplegia/

7 tetraplegi*.ti,ab.

8 diplegi*.ti,ab.

9 disabled children/

10 (disab* adj7 child*).ti,ab.

11 (handicap* adj3 child*).ti,ab.

12 or/1-11

13 exp Overweight/

14 Body Weight/

15 (obes$ or overweight or over-weight).tw.

16 exp Body Weight Changes/

17 (weight adj2 (loss or lost or losing or reduc$)).tw.

18 (weight adj2 (gain$ or increas$)).tw.

19 exp body fat distribution/ or body mass index/ or skinfold thickness/ or waist-hip ratio/

20 (body weigh$ or bodyweigh$ or body mass$ or bodymass or body fat$ or bodyfat$).tw.

21 Overnutrition/

22 (overeat$ or over-eat$ or overnourish$ or over-nourish$ or overnutrit$ or over-nutrit$).tw.

23 or/13-22

24    11 and 23

25 (risk or mortality).mp. or cohort.tw.

26 24 and 25

27 exp Pregnancy/ or pregnancy.mp

28 fetal development.mp or exp fetal development/

29 (fetal development or pregnancy or pregnan* or maternal or transplacental exposure* or gestat* or fetal development or fetal programming or fetal growth or foetal programming or foetal growth or gestational age or fetal age or foetal age or prenatal* or antenatal* or antepart*).tw.

30 27 or 28 or 29

31 26 and 30

**Web of Science**

TS=(fetal development or pregnancy or pregnan* or maternal or transplacental exposure* or gestat* or fetal development or fetal programming or fetal growth or foetal programming or foetal growth or gestational age or fetal age or foetal age or prenatal* or antenatal* or antepart*)

TS=(overweight or body weight or body weight changes or body mass index or body fat distribution or skinfold thickness or waist-hip ratio or overnutrition or obes* or over-weight or body weigh* or bodyweigh* or body mass* or bodymass* or body fat* or bodyfat* or overeat* or over-eat* or overnourish* or over-nourish* or over-nutri* or overnutri* or weight)

TS=(cerebral palsy or cerebral pals* or spastic* or CP or tetraplegi* or disabled children or quadriplegia)

TS=(risk or mortalit* or cohort)

**Supplement 2. Newcastle - Ottawa Quality Assessment Scale results for case-control** studies

| Question | **Option** | Nielsen,LF.2008^29^ | McPherson, J. A, 2016^12^ | Walstab,J. 2002^21^ |
| --- | --- | --- | --- | --- |
| Is the case definition adequate? | a) yes, with independent validation *****  b) yes, eg record linkage or based on self reports  c) no description | a | a | a |
| Representativeness of the cases | a) consecutive or obviously representative series of cases *****  b) potential for selection biases or not stated | a | b | b |
| Selection of Controls | a) community controls *****  b) hospital controls  c) no description | b | b | b |
| Definition of Controls | a) no history of disease (endpoint)*****  b) no description of source | a | a | b |
| Comparability of cases and controls on the basis of the design or analysis | 1. study controls for (Select the most important factor.***** 2. study controls for any additional factor (This   criteria could be modified to indicate specific  control for a second important factor.) ***** | a | a | a |
| Ascertainment of exposure | a) secure record (eg surgical records)*****  b) structured interview where blind to case/control status*****  c) interview not blinded to case/control status  d) written self report or medical record only  e) no description | a | a | a |
| Same method of ascertainment for cases and controls | a) yes*****  b) no | a | a | a |
| Non-Response rate | a) same rate for both groups*****  b) non-respondents described  c) rate different and no designation | b | a | b |

**Supplement 3. Newcastle - Ottawa Quality Assessment Scale results for cohort studies**

| Question | **Option** | Love,E. 2012^22^ | Crisham Janik,  2013^19^ | Pan, C, 2014^9^ | Forthun, I, 2016^20^ | Villamor, E,2017^13^ |
| --- | --- | --- | --- | --- | --- | --- |
| Representativeness  of the exposed cohort | 1. truly representative of the average (describe) in the community * 2. somewhat representative of the average in the community* 3. selected group of users eg nurses, volunteered) no description of the derivation of the cohort | b | b | b | b | b |
| Selection of the  Non-exposed cohort | 1. drawn from the same community as the exposed cohort* 2. drawn from a different source 3. no description of the derivation of the non-exposed cohort | a | a | a | a | a |
| Ascertainment of exposure | a) secure record (eg surgical records)*  b) structured interview*  c) written self report  d) no description | a | a | a | b | a |
| Demonstration that outcome of interest was not present at start of study | a) yes*  b) no | a | a | a | a | a |
| Comparability of cohorts on the basis of the design or analysis | 1. study controls for (select the most important factor)*   b) study controls for any additional factor* | a | a | a | a | a |
| Assessment of outcome | a) independent blind assessment*  b) record linkage*  c) self report  d) no description | b | b | b | b | b |
| Was follow-up long enough  for outcomes to occur | a) yes*  b) no | b | b | a | a | a |
| Adequacy of follow up  of cohort | 1. complete follow up - all subjects accounted for* 2. subjects lost to follow up unlikely to introduce bias - small number lost - > % (select an adequate %) follow up, or description provided of those lost)   c) follow up rate < % (select an adequate %) and no description of those lost  d) no statement | d | d | d | d | a |

**Supplement 4. Egger's test for publication bias test BMI≥40 kg/m^2^**

Tests for Publication Bias

Begg's Test

adj. Kendall's Score (P-Q) = 1

Std. Dev. of Score = 1.91

Number of Studies = 3

z=0.52

Pr > |z| = 0.602

z = 0.00 (continuity corrected)

Pr > |z| = 1.000 (continuity corrected)

Egger's test

---------------------------------------------------------------------------------------

Std_Eff | Coef. Std. Err. t P>|t| [95% Conf. Interval]

----------------------------------------------------------------------------------------

slope | -.2776831 .6100236 -0.46 0.728 -8.028768 7.473402

bias | .5506747 2.37846 0.23 0.855 -29.67053 30.77188

---------------------------------------------------------------------------------------

**Supplement 5. Egger's test for publication bias test BMI≥30 kg/m^2^**

Tests for Publication Bias

Begg's Test

adj. Kendall's Score (P-Q) = -2

Std. Dev. of Score = 2.94

Number of Studies = 4

z = -0.68

Pr > |z| = 0.497

z = 0.34 (continuity corrected)

Pr > |z| = 0.734 (continuity corrected)

Egger's test

------------------------------------------------------------------------------

Std_Eff | Coef. Std. Err. t P>|t| [95% Conf. Interval]

-------------+----------------------------------------------------------------

slope | .4447161 .7633778 0.58 0.619 -2.839834 3.729266

bias | -3.175011 2.014033 -1.58 0.256 -11.84069 5.490673

------------------------------------------------------------------------------

**Supplement 5. Begg's Test for publication bias test BMI18.5-24.9kg/m^2^, 25-29.9 kg/m^2^**

Tests for Publication Bias

Begg's Test

adj. Kendall's Score (P-Q) = 3

Std. Dev. of Score = 1.91

Number of Studies = 3

z = 1.57

Pr > |z| = 0.117

z = 1.04 (continuity corrected)

Pr > |z| = 0.296 (continuity corrected)

Egger's test

------------------------------------------------------------------------------

Std_Eff | Coef. Std. Err. t P>|t| [95% Conf. Interval]

-------------+----------------------------------------------------------------

slope | -2.946828 .6160239 -4.78 0.131 -10.77415 4.880498

bias | 5.665503 1.854189 3.06 0.201 -17.8942 29.22521

------------------------------------------------------------------------------
